# Elevated Levels of Active GSK3β in the Blood of Patients with Myotonic Dystrophy Type 1 Correlate with Muscle Weakness

**DOI:** 10.3390/ijms262110760

**Published:** 2025-11-05

**Authors:** Katherine Jennings, Cuixia Tian, Rebeccah L. Brown, Paul S. Horn, Benedikt Schoser, Hani Kushlaf, Nikolai A. Timchenko, Lubov Timchenko

**Affiliations:** 1 Division of Neurology, Cincinnati Children’s Hospital, 3333 Burnet Ave., Cincinnati, OH 45229, USAcuixia.tian@cchmc.org (C.T.);; 2 Department of Pediatrics, University of Cincinnati, 2600 Clifton Ave., Cincinnati, OH 45221, USAhani.kushlaf@uc.edu (H.K.);; 3 Department of Surgery, Cincinnati Children’s Hospital, 3333 Burnet Ave., Cincinnati, OH 45229, USA; 4 Department of Neurology, Friedrich-Baur-Institute, LMU Clinics, Ludwig-Maximilians-University, Ziemssenstr., 80336 Munich, Germany; benedikt.schoser@med.uni-muenchen.de; 5 Department of Neurology & Rehabilitation Medicine, University of Cincinnati, 260 Stetson St., Cincinnati, OH 45219, USA

**Keywords:** Myotonic Dystrophy type 1, CTG repeats, GSK3β kinase

## Abstract

Myotonic Dystrophy type 1 (DM1) is a complex disease affecting multiple tissues, including skeletal and cardiac muscles, the brain and the eyes. DM1 results from an expansion of CTG repeats in the 3′ UTR of the *DMPK* gene. Previously, we described that the small-molecule inhibitor of GSK3β, tideglusib (TG), reduces DM1 pathology in DM1 cell and mouse models by correcting the GSK3β-CUGBP1 pathway, decreasing the mutant CUG-containing RNA. Respectively, clinical trials using TG showed promising results for patients with congenital DM1 (CDM1). The drug development in DM1 human studies needs specific and noninvasive biomarkers. We examined the blood levels of active GSK3β in different clinical forms of DM1 and found an increase in active GSK3β in the peripheral blood mononuclear cells (PBMCs) in patients with CDM1, juvenile DM1 and adult-onset DM1 vs. unaffected patients. The blood levels of active GSK3β correlate with the length of CTG repeats and severity of muscle weakness. Thrombospondin and TGFβ, linked to the TG-GSK3β pathway in DM1, are also elevated in the DM1 patients’ blood. These findings show that the blood levels of active GSK3β might be developed as a potential noninvasive biomarker of muscle weakness in DM1.

## 1. Introduction

Myotonic Dystrophy type 1 (DM1) is a multisystem genetic disorder caused by unstable CTG expansions in the 3′-UTR of the *DMPK* gene [1,2]. DM1 is characterized primarily by skeletal muscle defects, including myopathy, atrophy, myotonia and weakness. DM1 patients also develop cardiomyopathy and Central Nervous System (CNS) deficits, including brain atrophy, memory and executive decision problems. In addition, DM1 patients might have cataracts and insulin resistance. Most severe, congenital DM1 (CDM1) affects patients before or at birth and is characterized by increased mortality, delay in development and severe muscle weakness. We and others found that the expanded CTG repeats cause disease by producing mutant *DMPK* mRNA with expanded CUG repeats, which is toxic to cells [3,4,5,6]. The mutant CUG repeats disrupt the RNA metabolism in DM1 cells via CUG RNA-binding proteins. The main RNA-binding protein families, studied in DM1, are MBNL (muscleblind-like splicing factors) [5] and CUGBP proteins (CUGBP Elav-like family of proteins, also known as CELF) [3,4]. The mutant CUG repeats sequester MBNL1, reducing its activity in DM1 cells [5,6]. CUGBP1 binds to bases of CUG hairpins [6], and its stability increases in the presence of mutant CUG RNA [7,8]. Although CUGBP1 levels are elevated in DM1, its activity is diminished [9]. The alterations of CUGBP1 activity in DM1 occur due to an increase in active GSK3β kinase, which regulates CUGBP1 phosphorylation at Ser302 through the cyclin D3-CDK4 pathway [9]. While there are no disease-based therapies for DM1, the number of developing therapeutics for DM1 is rapidly growing. They include antisense oligonucleotides that target the mutant *DMPK* mRNA containing CUG repeats, and small molecules that affect targets downstream of CUG repeats, reducing disease severity and microRNAs [10,11,12,13,14,15,16,17,18]. Small-molecule inhibitors of GSK3β, including tideglusib (TG), correct the GSK3β-CUGBP1 pathway in DM1 and cause the degradation of the mutant CUG repeats [9,18]. The safety and efficacy of TG were evaluated in patients with CDM1 in Phase II and Phase III clinical trials with promising results, leading to the preparation of a new Phase III clinical trial for adult-onset DM1 [19,20,21]. There are several developing biomarkers for DM1. Among them is the Splicing Index (SI), which summarizes splicing changes for several mRNAs in DM1 patients [22,23,24]. Since the inhibitor of GSK3β was used in the DM1 clinical trials, we examined if measuring GSK3β levels in DM1 blood samples may correlate with muscle weakness and serve as an indicator of DM1 severity. Previously, we described the pathological increase in active (P-Y216)-GSK3β (also called active GSK3β) in skeletal muscle biopsies and primary myoblasts from patients with adult-onset DM1 and CDM1 [9,18]. However, monitoring the correction of GSK3β in skeletal muscle biopsies during clinical trials is difficult because of the invasive and painful nature of collecting muscle biopsies, especially in children [25,26]. Therefore, we examined whether the pathological increase in active GSK3β could be detected in blood samples of patients with DM1 and if this increase in GSK3β correlates with muscle weakness in patients with DM1.

## 2. Results

### 2.1. The Levels of Active GSK3β Are Increased in the Blood of Patients with Adult-Onset DM1 and Correlate with CTG Repeat Number

**Figure 1:**
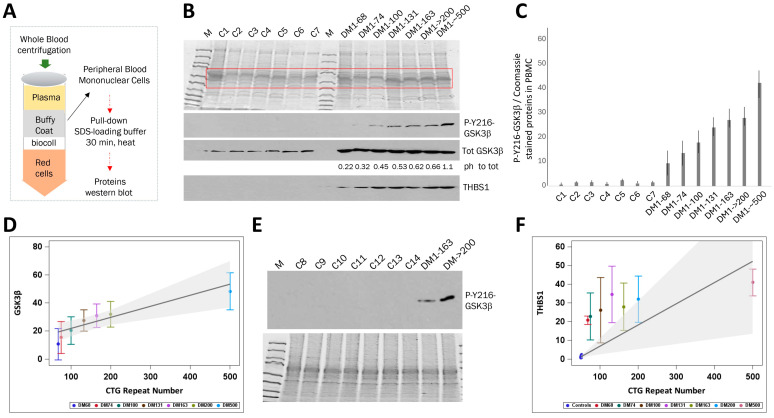
Levels of active GSK3β are increased in PBMCs from patients with adult-onset DM1 relative to unaffected control patients. (**A**) The scheme shows the preparation of PBMC proteins for Western blot analyses. The whole blood samples were separated by centrifugation in the gradient of Biocoll. The buffy coats were collected, and PBMCs were spun down. The cell pellets were resuspended in SDS-loading buffer, boiled for 30 min, and analyzed by Western blot assay as described in the Methods section. (**B**) Western blot analyses of proteins from PBMCs from control and adult-onset DM1 patients. Control samples 1–3 are familial controls not affected by DM1 (a 56-year-old female, a 49-year-old male and 55-year-old male, respectively). Control samples 4–7 are not familial participants (a 59-year-old female, a 35-year-old female, a 66-year-old male and 28-year-old female). The labels for DM1 samples include the length of CTG repeats. The DM1–68, DM1–74, DM1–100, DM1–131, DM1–163, DM1–>200 and DM1–~500 are samples from participants with adult-onset DM1 (females of 48, 43, 59, 31, 40, 29 and 40 years, respectively). Coomassie blue staining of the proteins extracted from PBMCs is shown on the top. Active GSK3β, total GSK3β and THBS1 were measured. Ph to tot: numbers below the total GSK3β image represent the ratios of the active GSK3β signals to total GSK3β in the same DM1 blood samples. (**C**) Bar graphs of the active GSK3β signals shown in (**B**) relative to Coomassie signals. Standard deviations (SDs) calculated from three repeats are shown. (**D**) Weighted regression results of active GSK3β levels on CTG repeat number, using data from 1B. For each DM1 subject, the dependent variable is the mean active GSK3β, and the weight is the inverse of the variance. Predicted GSK3β is shown with shaded 95% confidence bands. Standard deviation bars are calculated from three replicates. Colors denote the patients. (**E**) Western blot analysis of additional unrelated control blood samples vs. DM1 samples with antibodies to active GSK3β. C8, C10, C11, C13 and C14 are females of 41, 28, 38 and 52 years of age, respectively. C9 and C12 are males of 69 and 62 years. DM1 samples from patients with 163 and >200 CTG repeats analyzed in (**B**) are used as positive controls. Coomassie staining of the proteins extracted from PBMCs is shown on the bottom. (**F**) Using the same approach applied to GSK3β, a weighted regression was performed with THBS1 levels from 1B as the dependent variable and CTG repeat number as a predictor. For control samples, the maximum limit of 50 CTG repeats was applied because the exact number of repeats in these unaffected controls is not known. Bars indicate THBS1 standard deviations from three repeats. Predicted THBS1 is shown with shaded 95% confidence bands.

In our previous studies on GSK3β expression in DM1 skeletal muscle, we observed increased total GSK3β, a strong reduction in inactive (S9-phosphorylated) GSK3β and elevation of active GSK3β [9]. Therefore, in current study, we measured the levels of active GSK3β in DM1 PBMCs from adult-onset DM1 patients with varying numbers of CTG repeats (from 68 to ~500 repeats). To identify possible changes in GSK3β levels in the blood samples of these patients, we enhanced the sensitivity of GSK3β detection in PBMCs by pulling the PBMCs from buffy coats and extracting proteins using the improved protein loading buffer (Figure 1A). Immunoanalysis of these DM1 blood samples showed that the levels of active GSK3β are increased in DM1 patients compared to control participants (Figure 1B,C and Figure S1). Note that Figure 1B shows relative amounts of active GSK3β in the PBMCs from control and DM1 patients. To quantify the levels of GSK3β, smaller amounts of proteins (about 10 μg) were used for Western blot assay, and the intensity of the signals was analyzed using ImageJ software (ImageJ v2). Under these conditions, the level of active GSK3β was slightly above the background in control samples, while GSK3β was increased in PBMCs of DM1 patients. The weak GSK3β signal in control samples reflects the loading of low amounts of total proteins (10 μg). Using higher amounts of protein allows detection of GSK3β in PBMCs from control patients, but the signal then exceeds the quantitative range. Thus, the findings shown in Figure 1B,C indicate that the levels of active GSK3β are increased in the PBMCs from adult-onset DM1 patients.

The levels of active GSK3β in PBMCs rose from ~7 times higher in the patient with 68 CTG repeats compared to controls to ~30 times higher in a patient with ~500 CTG repeats (Figure 1B,C). There was also a marked difference in the levels of active GSK3β between patients with DM1 carrying 74 and 100 CTG repeats vs. those in a patient with ~500 CTG repeats (Figure 1B,C). Using this protocol, we observed an increase in the GSK3β between DM1 patients with a relatively small difference in the CTG repeat length in the range from 68 to 163 repeats (Figure 1C). Regression analysis suggested a potential link between CTG repeat length and active GSK3β levels in DM1 blood samples (Figure 1D). This analysis showed that the active GSK3β rises by 1.6 for every 10-unit increase in CTG repeat length (*p* = 0.0004). Thus, active GSK3β is increased in the blood of patients with adult-onset DM1, and this increase is linked to the number of CTG repeats present. There was no correlation between the levels of active GSK3β and patients’ age. For instance, low levels of GSK3β were observed in patients of 44, 48 and 59 years of age (average 50 years), while the higher levels of GSK3β were in patients of 29, 31 and 40 years of age (average 33 years). To confirm the increase in active GSK3β in DM1 blood, GSK3β levels were examined in PBMCs from additional unrelated control patients which were recruited later in the study. As shown, the inclusion of additional control samples confirmed the increase in active GSK3β in the blood of DM1 patients (Figure 1E).

To address whether the observed increase in active GSK3β in DM1 blood samples parallels that seen in DM1 skeletal muscle biopsies, we measured the total GSK3β in control and DM1 blood samples. As shown in Figure 1B, the total GSK3β is increased in all analyzed DM1 blood samples compared to controls. These data indicate that, as with DM1 skeletal muscle [9], DM1 PBMCs have increased total and active GSK3β. However, total GSK3β levels are not affected by the length of CTG repeats.

Previously, we found that the expression of mutant CUG repeats mis-regulated several pathways in the skeletal muscle of DM1 mice (*HSA^LR^* model), including pathways regulating transport, secretion, synaptic vesicle localization, synaptic vehicle cycle, system development and cell differentiation [27]. Treatment of *HSA^LR^* mice with TG corrected a group of abnormally expressed genes in *HSA^LR^* muscle, including chloride channel 1, which is associated with myotonia [28,29], and a circulating glycoprotein, thrombospondin 1 (THBS1). These findings suggest that the TG-GSK3β pathway targets chloride channel 1 and THBS1 in DM1. THBS1 participates in multiple cellular processes due to interactions with other proteins and extracellular matrix components [30,31]. THBS1 interacts with and activates transforming growth factor β (TGFβ), which is associated with the induction of muscle atrophy and fibrosis by promoting expression of collagens [32,33]. The increase in Thbs1 in *HSA^LR^* muscle correlates with the development of muscle atrophy (a loss of fibers) in these mice. We found that Thbs1 is also increased in the skeletal muscle of S302A-CUGBP1 knock-in mice in which the S302 (in mice, S333) site of phosphorylation controlled by the GSK3β-cyclin D3-CDK4 was mutated. The increase in Thbs1 in *HSA^LR^* and S302A-CUGBP1 KI mice suggests that the GSK3β-CUGBP1 signaling controls *Thbs1* [27]. The Thbs1 levels were corrected in *HSA^LR^* mice treated with TG. To determine if THBS1 protein, as a downstream component of the TG-GSK3β pathway, is also elevated in DM1 patients, we analyzed THBS1 protein levels in PBMCs from patients with adult-onset DM1. This analysis showed that the blood levels of THBS1 are also increased in DM1 patients compared to controls (Figure 1B,F and Figure S2). Regression analysis indicated that every 10-unit rise in CTG repeats increased THBS1 levels in DM1 blood by 1.1 (*p* = 0.014) (Figure 1F). To summarize, our findings indicate that the levels of active GSK3β are higher in blood samples from adult-onset DM1 patients and correlate with CTG repeat number.

### 2.2. The Increase in Active GSK3β in PBMCs from Adult-Onset DM1 Patients Correlates with Muscle Weakness

**Figure 2:**
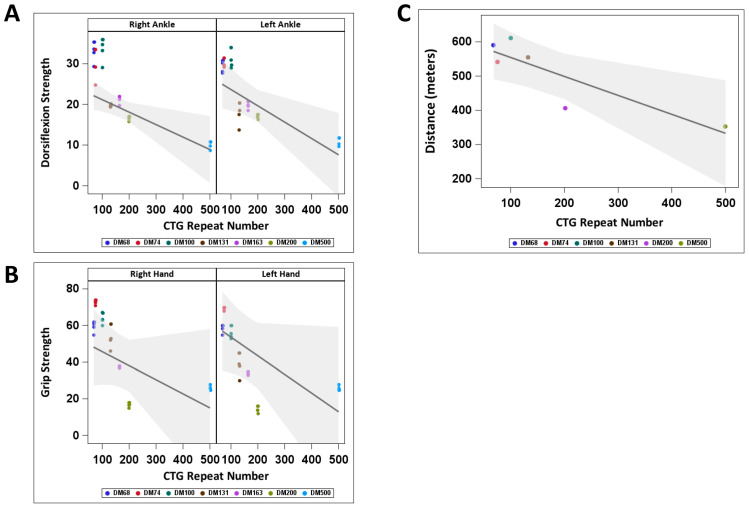
Skeletal muscle function in a group of patients with adult-onset DM1 with increased levels of active GSK3β in PBMCs. Outcomes from the weighted regression analyses of dorsiflexion (**A**) and grip strength (**B**) were assessed in relation to CTG repeat number. Each subject’s mean is the dependent variable, weighted by the inverse variance. The 6MWT data (**C**) are presented without weighting adjustments. Predicted dorsiflexion strength (**A**), grip strength (**B**) and distance in 6MWT (**C**) are shown with 95% confidence bands. Colors show DM1 patients.

To determine if the increase in active GSK3β in the blood samples of patients with adult-onset DM1 correlates with muscle weakness, we examined the skeletal muscle performance in the group of DM1 patients, in which GSK3β levels were evaluated (Figure 1B,C), by measuring the grip strength, ankle dorsiflexion strength, manual strength and walking distance during the 6 min test. As shown in Figure 2, DM1 patients with short (68–100) CTG repeats showed approximately similar muscle performance. However, DM1 patients with 163–500 CTG repeats showed reduced left and right ankle strength, grip strength and distance in the 6 MW test (Figure 2A–C). The dorsiflexion strength was closely linked to the number of CTG repeats, with each increase of 10 in CTG length resulting in decrease of 0.3 for the right ankle and 0.4 for the left ankle (*p* = 0.023; *p* = 0.024).

**Figure 3:**
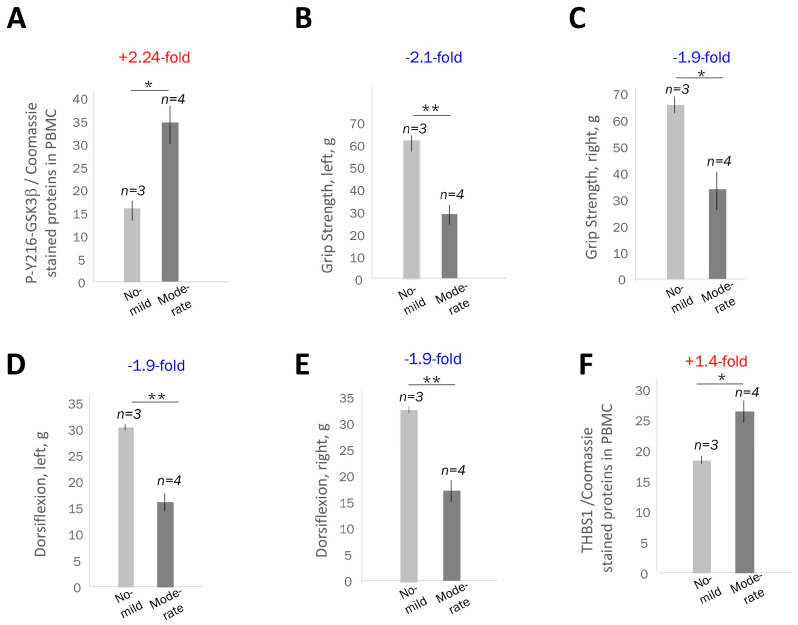
Correlation of the average levels of active GSK3β in blood with the muscle performance of DM1 patients with different severity of muscle disease. (**A**) Comparison of the average GSK3β levels in PBMCs from adult-onset DM1 patients with 68–100 CTG repeats without myotonia (n = 3) and in patients with 131–500 CTG repeats with myotonia and muscle weakness (n = 4). Western blot data and signal intensity analyses (using Image J2) are shown in Figure 1B,C. A group of DM1 patients with mild muscle disease includes DM1 patients: DM1–68, DM1–74 and DM–100. A group of DM1 patients with moderate muscle weakness includes DM1–131, DM1–163, DM1 > 200 and DM1 ~ 500. The values of grip strength (**B**,**C**) and ankle strength (**D**,**E**) were compared in adult-onset DM1 patients with mild (n = 3) and moderate (n = 4) skeletal muscle weakness. The skeletal muscle outcomes for each patient with DM1 are shown in Figure 2A,B. (**F**) Comparison of the blood levels of THBS1 in adult-onset DM1 patients with mild and moderate skeletal muscle disease. Western blot analyses of THBS1 in DM1 blood samples are shown in Figure 1B. Standard deviations and the number of DM1 patients is shown. * and ** are *p* values < 0.05 and <0.01 for the DM1 patients with mild vs. moderate muscle weakness.

Grip strength decreased by 0.8 (right hand) and 1.0 (left hand) for every 10 additional CTG repeats, but these changes were not statistically significant. A significant change was observed between 6 min distance and CTG repeat length, with each increase of 10 in CTG repeat length associated with a reduction of 5.5 in 6MWT distance (*p* = 0.024). Manual muscle strength was similar on the left and right sides of the body in this group of patients, with a minor strength reduction in a patient carrying 163 CTG repeats. These data show that muscle weakness assessed by ankle dorsiflexion and 6MWT tests is significantly associated with CTG repeat length, while grip strength tends to decrease as CTG repeats increase in adult-onset DM1 patients. To determine if the increase in active GSK3β in the PBMCs in patients with adult-onset DM1 correlates with the muscle weakness determined at the time of blood collection, we compared the average active GSK3β levels in adult-onset DM1 patients with no previously reported myotonia and mild muscle phenotype, based on the grip and ankle strength tests (Figure 2A,B), which carry 68–100 CTG repeats, with those in a group of DM1 patients showing moderate muscle symptoms including myotonia and weakness (patients with 131, 163, >200 and ~500 CTG repeats). As shown in Figure 3A, the mean GSK3β levels are ~2-fold increased in the PBMCs in the DM1 group with moderate muscle weakness relative to those in DM1 patients with shorter CTG expansions and mild muscle weakness.

Comparison of the muscle performance measures such as grip strength and ankle dorsiflexion strength in the DM1 groups with mild and moderate muscle weakness showed that there is also about a 1.9–2.1-fold reduction in strength in the left and right hands and left and right ankles (Figure 3B–E). Thus, there is a correlation between the increase in active GSK3β levels in adult-onset DM1 PBMCs and muscle weakness measured by the grip and ankle dorsiflexion strength. Note that this correlation was identified in adult-onset DM1 patients with mild and moderate muscle weakness using quantitative conditions of immunodetection of active GSK3β in PBMCs. The correlative analysis might be more complex in adult-onset patients with DM1, which show multisystemic disease involvement, with cardiac and metabolic problems, which might negatively affect skeletal muscle performance. We found that the levels of THBS1 increased ~1.4-fold in the PBMCs from adult-onset DM1 patients with moderate muscle weakness relative to those with no or mild weakness (Figure 3F). The ~1.4-fold increase in THBS1 in the DM1 blood samples shows an approximate correlation with the ~2-fold reduction in muscle strength in patients with adult-onset DM1.

### 2.3. Active GSK3β Is Increased in PBMCs of Patients with Juvenile and Congenital DM1 Relatively Controls, Correlating with Muscle Weakness

**Figure 4:**
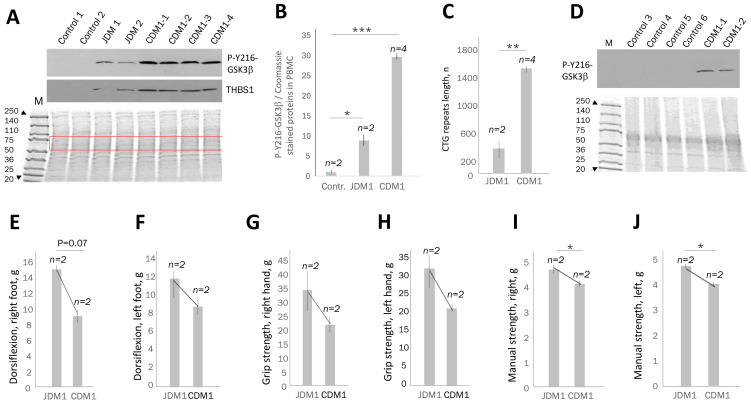
The levels of active GSK3β are increased in PBMCs of patients with juvenile and congenital DM1 correlating with muscle weakness. (**A**) Western blot analyses of the active GSK3β and THBS1 in PBMCs from the familial control patients (C1 and C2 are a 56-year-old female and a 49-year-old male, respectively), patients with JDM1 (17- and 19-year-old females) and CDM1 (CDM1-1–4 are an 18-year-old female, a 16-year-old male, a 5-year-old female and a 5-month-old female, respectively). Coomassie staining of the PBMC proteins is shown below the Western blot images. (**B**) The average active GSK3β levels in the PBMCs from control, JDM1 and CDM1 patients were adjusted to the Coomassie-stained proteins shown in the box in (**A**). * and *** are *p* values < 0.05 and <0.001 for the average GSK3β signal in JDM1 compared to control and for the average GSK3β in CDM1 patients vs. control. (**C**) Comparison of the mean length of CTG repeat expansions in JDM1 and CDM1 patients. ** is *p* value < 0.01 for JDM1 vs. CDM1. (**D**) Western blot analysis of active GSK3β in pediatric and adolescent control blood samples and CDM1 samples. Coomassie staining is shown on the bottom. The average values of ankle dorsiflexion (**E**,**F**), grip strength (**G**,**H**) and manual strength (**I**,**J**) in patients with JDM1 and CDM1 are shown. The number of patients per group is also shown. * indicates a *p* value < 0.05 for JDM1 vs. CDM1. While GSK3β levels were evaluated in blood samples from four patients with CDM1, the muscle performance was analyzed in two patients with CDM1 aged 16 and 18. No muscle performance data were collected in two CDM1 patients due to early age (0.5 and 5 years).

Next, we examined the active GSK3β levels in PBMCs from patients with JDM1 and CDM1 by Western blot analysis using the protocol applied to the analysis of adult-onset DM1 patients. Figure 4A,B and Figure S3 show that the levels of active GSK3β were increased in PBMCs from JDM1 (about 10-fold) and CDM1 (about 34-fold) patients relative to unaffected controls. THSB1 was also elevated in JDM1 and CDM1 blood samples (Figure 4A).

The increase in active GSK3β levels in PBMCs approximately correlates with ~3.9-times longer CTG expansions in CDM1 patients than in patients with JDM1 (Figure 4B,C). Thus, we conclude that the analyzed CDM1 and JDM1 blood samples show a strong (~10–34-fold) increase in active GSK3β vs. control blood samples, and this increase in GSK3β in blood correlates with the length of CTG repeats. To examine if the increase in active GSK3β in CDM1 blood is not due to age differences in control and CDM1 patients, the levels of active GSK3β were compared in unrelated control blood samples of pediatric patients not affected by DM1 and CDM1 patients. As shown in Figure 4D, signals of active GSK3β are detectable in blood of CDM1 patients of 16–18 years of age, but not in the control blood samples of patients of 10–18 years of age.

We next asked if the increase in active GSK3β in the blood samples of JDM1 and CDM1 patients correlates with muscle dysfunction. As shown, the ankle dorsiflexion strength (right foot) was ~1.6-fold weaker in CDM1 than in JDM1 patients, while the ankle dorsiflexion strength of the left foot was ~1.33-fold weaker in CDM1 than in JDM1 (Figure 4E,F). The grip strength of the right hand was ~1.6-fold lower in CDM1 patients than in JDM1 (Figure 4G), and the strength of the left hand was approximately 1.5-fold reduced in CDM1 compared to JDM1 (Figure 4H). The severity of muscle weakness, as examined by the manual test, also correlated with increased GSK3β levels in CDM1 relative to JDM1. For instance, the manual muscle strength of both right- and left-side muscle groups was ~17% less in CDM1 than in JDM1 (Figure 4I,J). Thus, the increase in the levels of active GSK3β in the PBMCs correlates with muscle weakness in patients with JDM1 and CDM1.

### 2.4. PBMC Samples from Patients with DM1 Might Be a Source for Evaluation of Proteins Connected to the GSK3β-CUGBP1 Pathway in DM1

**Figure 5:**
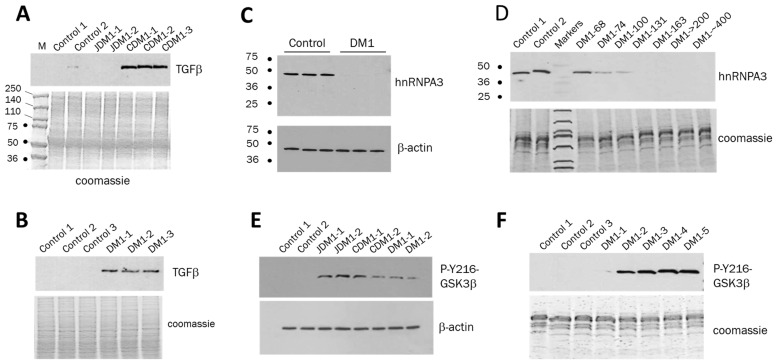
TGFβ is increased in PBMCs of patients with CDM1 (**A**) and DM1 (**B**). PBMC samples from controls, patients with CDM1, JDM1 and adult-onset DM1 were analyzed by Western blot with Abs to TGFβ. Coomassie staining on the bottom shows integrity and equal protein loading. PBMCs were from two JDM1 samples and three CDM1 samples, described in Figure 4. PBMCs from three patients with adult-onset DM1 carry 74, 100 and about 500 CTG repeats. (**C**) hnRNP-A3 is reduced in human myoblasts derived from patients with DM1. Western blot analysis was performed with antibodies to hnRNP-A3 and β-actin (control for protein loading) using human myoblasts from three normal controls and three patients with adult-onset DM1. (**D**) hnRNP-A3 is reduced in PBMCs from adult-onset patients with DM1 which carry >100 CTG repeats. PBMC samples from controls (n = 2) and patients with adult-onset DM1 (n = 7) were analyzed by Western blot with antibodies to hnRNP-A3. Coomassie staining of the protein samples is shown at the bottom. (**E**) Active GSK3β is increased in human fibroblasts from patients with DM1. Western blot analysis used human fibroblast cell lines derived from two controls, two JDM1, two CDM1 and two adult-onset DM1 patients with antibodies to pY216-GSK3β and β-actin (control for protein loading). JDM1 and CDM1 fibroblasts were derived from the same JDM1 and CDM1 patients described in Figure 4. Fibroblasts from two adult-onset DM1 patients carry 74 and 100 CTG repeats. (**F**) Active GSK3β is increased in PBMCs from adult-onset DM1 patients without genetic confirmation. Coomassie staining shows equal protein loading. Patient DM1-1 has no muscle phenotype, while the remaining patients have muscle weakness and myotonia.

A strong increase in active GSK3β in DM1 blood samples suggested that levels of some proteins involved in the GSK3β-CUGBP1 pathway might also be changed in DM1 blood samples. We found that one of the proteins corrected by TG in DM1 mice, THBS1, was increased in DM1 blood samples (Figure 1B,F). It has been shown that THBS1 binds to and activates a secreted protein, transforming growth factor β1, TGFβ1, which plays a key role in regulating cell growth and differentiation [32]. Activation of TGFβ induces fibrosis in skeletal muscle and other tissues by promoting the expression of collagens. Thus, the increase in THBS1 in the blood samples of patients with adult onset of DM1 suggested a potential elevation of TGFβ in DM1. Therefore, we evaluated the levels of TGFβ in blood samples from patients with JDM1, CDM1 and adult-onset DM1. As shown, TGFβ levels are strongly increased in the PBMCs from patients with CDM1 (Figure 5A) and adult-onset DM1 (Figure 5B), but not JDM1 (Figure 5A).

Identifying the increase in TGFβ in CDM1 and adult-onset DM1 is important, since it has been shown that TGFβ is increased in DMD, associated with reduced muscle degeneration and the development of fibrosis [33,34]. The increase in TGFβ in the blood samples from patients with CDM1 and adult-onset DM1 indicates degenerative processes in DM1 muscle. It remains to be determined whether TGFβ levels in blood correlate with the severity of muscle weakness in DM1 by analyzing additional patients with DM1. The reason for the lack of increase in TGFβ in JDM1 blood remains to be investigated. In addition to the studies described above, the PBMC samples from DM1 patients could also help evaluate proteins which might be connected to the degradation of mutant CUG repeats. In this regard, we recently identified a set of mRNAs altered in the brains of CUGBP1-S302A-KI mice (own unpublished data) in which accumulation of inactive CUGBP1 (CUGBP1^REP^) mimicking increased amounts of CUGBP1^REP^ in DM1 tissues causes severe brain atrophy [27]. Among altered genes, a set of mRNAs with reduced expression in the cortices of CUGBP1-KI mice includes mRNA encoding RNA-binding protein hnRNP-A3. This protein is of great interest to our studies because of its known role in other neurodegenerative diseases. First, hnRNP-A3 is associated with ALS and FTLD [35,36], where it binds to expanded GGGGCC repeats. Second, the reduction in hnRNP-A3 in FTLD fibroblasts increases the accumulation of toxic RNA foci, suggesting that the decrease in hnRNP-A3 might increase the stability of the toxic RNAs [35]. Third, the reduction in hnRNP-A3 mRNA in CUGBP1-KI brain cortices (own unpublished data) suggests that this protein might be reduced in DM1 tissues, since the amounts of inactive CUGBP1, unphosphorylated at S302, are increased in DMSXL mice expressing > 1,000 CTG repeats [18]. Therefore, we examined hnRNP-A3 expression in DM1 myoblasts and found that hnRNP-A3 is undetectable in primary myoblasts derived from patients with DM1. In contrast, hnRNP-A3 was observed in normal myoblasts (Figure 5C). Since hnRNP-A3 is also expressed in PBMCs, we evaluated hnRNP-A3 levels in PBMCs from adult-onset DM1 patients and found a reduction in hnRNP-A3 in DM1 patients carrying more than 100 CTG repeats (Figure 5D). The reduction in hnRNP-A3 in DM1 might contribute to the accumulation of CUG foci.

The evaluation of the proteins of the GSK3β-CUGBP1 pathway in the blood of DM1 patients raised the question of whether the levels of active GSK3β in DM1 blood are comparable to those in other cells like skin fibroblasts. We compared the levels of active GSK3β in primary fibroblasts from the same patients used to examine GSK3β levels in PBMC samples vs. controls and found the same trend showing an increase in active GSK3β in DM1 fibroblasts (Figure 5E). During patients’ recruitment for the GSK3β study, we found that some patients with adult-onset DM1 had a definite DM1 phenotype without genetic confirmation. We evaluated the active GSK3β levels in this group of DM1 patients and found an increase in active GSK3β in their PBMCs (Figure 5F). Note that the GSK3β signal in a patient DM1-1 without muscle phenotype based on the clinical information and the grip and ankle strength tests at the time of the blood sample collection is very weak and barely seen on the film; however, other DM1 patients in this group have muscle phenotypes including myotonia (DM1-2 and DM1-3), muscle atrophy (DM1-2, DM1-3 and DM1-4) and muscle weakness (patients DM1-2, DM1-3, DM1-4 and DM1-5). Respectively, the levels of active GSK3β are increased in PBMCs of these patients. Thus, the increase in active GSK3β is detected in PBMCs of adult-onset patients with apparent DM1 phenotype but without genetic confirmation.

## 3. Discussion

The first important result of this study is that the levels of active GSK3β are increased in all three clinical forms of DM1, including patients with CDM1, JDM1 and adult-onset DM1. We have not identified even a single unaffected patient with increased levels of active GSK3β comparable to those in DM1 patients; however, the number of analyzed control samples should be increased. The second result described in this manuscript is that the increase in the levels of active GSK3β is detected in PBMCs of DM1 patients. While GSK3β is detected in the buffy coats in controls and DM1 patients without pelleting PBMCs, we found that the quantitative analysis of proteins is more sensitive if PBMCs are spun down from the buffy coats. Also, using lesser amounts of total proteins (10 μg per lane) from PBMCs increases the sensitivity of the GSK3β quantification. Identification of the active GSK3β increase in PBMCs in DM1 patients allows for the detection of pathological GSK3β elevation in blood samples, eliminating the need for muscle biopsies in clinical studies. However, the GSK3β assay in the blood of DM1 patients may need to be simplified for clinical use by developing ELISA. It appears that the pathological levels of active GSK3β in PBMCs in DM1 patients correlate with the length of CTG repeats. As shown in Figure 1B–D, the levels of active GSK3β increase in PBMCs when the length of CTG repeats grows from 68 to 200 CTG repeats, and they grow even more in the patient with about 500 CTG repeats. This result suggests that the elevation of active GSK3β in DM1 might depend on the DM1 mutation—the length of CTG repeats. The most important result of this study is that there is a correlation between the levels of active GSK3β in PBMCs in adult-onset DM1 patients with muscle weakness. We found that the muscle strength is reduced by about 2-fold in adult-onset DM1 patients who carry from 131 to 500 CTG repeats compared to DM1 patients with a short range of CTG repeats from 68 to 100 CTG repeats. The ~2-fold reduction in muscle strength correlated with a ~2-fold increase in active GSK3β in PBMCs in the same patients with DM1 (Figure 3A–E). These data show that the detection of a pathological increase in active GSK3β in blood samples from patients with DM1 could be developed as a noninvasive biomarker of muscle weakness in DM1. However, to determine if the elevation of active GSK3β in the blood of DM1 patients might be used as a biomarker of muscle weakness, additional studies analyzing the levels of active GSK3β in the same patients over time are required. This is the limitation of the current study. Since CTG expansions in DM1 skeletal muscle are longer than in blood, the muscle levels of active GSK3β should also be correlated with the length of CTG repeats in skeletal muscle. Our findings show the same trend of an increase in active GSK3β in fibroblasts derived from patients with DM1 (Figure 1B,C and Figure 5E). However, additional studies are needed to correlate GSK3β levels in DM1 blood and skeletal muscle. Regardless of these questions, we showed that the blood levels of active GSK3β correlate with muscle strength in adult-onset DM1. As noted above, this correlation might be more complex if DM1 patients, in addition to skeletal muscle phenotype, also have metabolic and cardiac abnormalities, which might affect skeletal muscle performance. In addition, we found that the comparison of GSK3β levels with the muscle function should be conducted separately for CDM1/JDM1 and adult-onset DM1 groups.

The findings described in this paper suggest that the blood levels of active GSK3β could be used to monitor GSK3β correction with TG or other small-molecule inhibitors of GSK3β in DM1 clinical trials. Tracking the blood levels of active GSK3β in DM1 could also be applied in DM1 clinical trials using other developing therapeutics such as ASOs and small molecules that directly target the mutant *DMPK* mRNA. Since the TG-GSK3β pathway reduces the mutant *DMPK* mRNA in human DM1 cells and CUG-containing transcripts in *HSA^LR^* muscle [18], the increased levels of active GSK3β in PBMCs in DM1 patients could be used as one of the potential biomarkers to monitor the effects of these therapeutics. Interestingly, two proteins downstream of the TG-GSK3β pathway, as follows from the studies of skeletal muscle of *HSA^LR^* mice [27], THBS1 and TGFβ, also increased in PBMCs in DM1 patients. The levels of THBS1 correlate with muscle weakness in DM1 patients. While quantification of TGFβ levels in PBMCs from DM1 patients with different disease severity needs further study, TGFβ is strongly increased in CDM1 and adult-onset DM1 relative to control samples. Besides GSK3β, THBS1 and TGFβ, other key proteins related to DM1 pathogenesis could be tracked in DM1 blood during clinical trials. As shown in Figure 5D, the collected PBMC samples from patients with DM1 can serve as a source for evaluating the expression of proteins which might be related to the degradation of mutant CUG repeats. It is possible that hnRNP-A3 could be a candidate protein contributing to the accumulation of CUG foci in DM1. If reducing hnRNP-A3 increases toxic CUG foci, therapeutic effects on degrading CUG repeats can be analyzed by monitoring hnRNP-A3 levels in DM1 blood samples. It remains to be shown whether TG can correct the levels of hnRNP-A3 in DM1 cells, improving the degradation of toxic CUG-containing RNA. While the increase in active GSK3β in PBMCs from patients with DM1 correlates with muscle weakness (Figure 3), it is also essential to determine if the pathological increase of active GSK3β in PBMCs correlates with the severity of brain atrophy in DM1 and with other symptoms such as cardiac defects and insulin resistance. These critical questions should be addressed in future studies.

## 4. Materials and Methods

### 4.1. Human Subjects

The IRB at CCHMC approved the human study protocol. All participants (or legal guardians or parents) provided written consent forms. Twenty unaffected participants, both male and female, aged 25 to 55, included three familial and 17 non-familial controls. Four whole blood samples (males) from unaffected patients (no muscle disease) aged 10 to 18 years were discarded samples from anonymous patients. Data on CTG repeat length, myotonia and neuropsychological evaluation were gathered from DM1 participants’ medical records. The muscle performance was assessed at the time of the study visit as described below. The group of DM1 participants included patients with CDM1 (males and females aged 5 months to 18 years with ~1250–1700 CTG repeats) and patients with JDM1 (females, aged 17 to 19, with >150–626 CTG repeats). A group of 12 patients with adult-onset DM1 included seven DM1 patients in which DM1 diagnosis was confirmed by genetic testing (females, aged 31 to 59 with the 68 to about 500 CTG repeats). The DM1 group also included patients who showed the clinical phenotype of DM1 including myotonia and muscle weakness (males and females aged 32 to 73) without genetic test confirmation.

### 4.2. Muscle Performance Assessments

The muscle performance assessments were performed during the study visit following the collection of vital signs (height, weight, temperature, blood pressure and heart rate). Skeletal muscle performance was assessed using grip strength, ankle dorsiflexion strength, manual strength and a 6 min walk test. Grip strength and ankle dorsiflexion strength were measured from both hands and ankles, with averages calculated from four to five measurements. The manual strength evaluation was performed from the right and left sides from the following areas: shoulder abduction, elbow flexion, elbow extension, wrist extension, wrist flexion, finger grip, finger abduction, finger extension, hip flexion, knee extension, knee flexion, ankle dorsiflexion and ankle plantar flexion; the evaluation was performed using the rating scale.

### 4.3. Skin Biopsy and Generation of Fibroblasts

The skin was cleaned with an alcohol-based solution, dried and numbed with lidocaine. A small piece of the top layer of skin was cut out using a 2 mm round Visipunch instrument. The skin sample was placed into a sterile tube containing cell culture medium (DMEM (Gibco, Life Technologies, Grand Island, NY, USA), 20% FBS (HyClone, Life Technologies, Grand Island, NY, USA) and 1% penicillin/streptomycin (Gibco, Life Technologies, Grand Island, NY, USA) for the preparation of fibroblast cells. Pressure was applied to the place of the biopsy to stop bleeding; a small amount of antibiotic ointment was added to the place of the biopsy, and the site was bandaged. Skin samples were placed in the 6 cm cell culture dish coated with collagen. Cells from human tissue were expanded in a medium with DMEM, 20% FBS and 1% penicillin/streptomycin and maintained in a medium with DMEM, 10% FBS and 1% penicillin/streptomycin.

### 4.4. Blood Collection and Preparation of Protein Extracts from PBMCs

A 2 mL venous blood sample was collected in a tube containing dipotassium EDTA (BD, Becton, Dickinson, and Company, Franklin Lakes, NJ, USA). The PBMCs were extracted from the whole blood samples after blood collection without freezing or keeping samples at 4 °C. Biocoll separating solution (7 mL) was added to 15 mL sterile plastic tubes to separate 1 mL of the whole blood diluted to 2 mL with sterile 1× PBS (1:1). Equal parts of blood and sterile PBS were mixed in a separate sterile tube and carefully applied over Biocoll solution. Tubes were centrifuged at 1200× *g* for 20 min at 16 °C with the brakes and acceleration turned off to prevent gradient disturbance. Plasma and the PBMCs were collected after the separation. The PBMCs were spun down, and the cell pellets were resuspended in the modified protein loading buffer, which included 2% SDS and 10 mM β-mercaptoethanol. The resuspended cells were boiled for 30 min and spun down at 10,000 rpms for 10 min. The supernatant, containing total PBMC proteins, was subjected to gel electrophoresis, and the gel was stained by Coomassie staining to examine the integrity of the proteins. After this step, equal amounts of the proteins were used for Western blot analyses as described below.

### 4.5. Myoblast and Fibroblast Cell Culture

Human myoblasts were maintained in an F10 medium (Sigma-Aldrich, Saint Loius, MO, USA) containing 1% sodium bicarbonate (Gibco, Life Technologies, Grand Island, NY, USA), 15% fetal bovine serum (HyClone, Life Technologies, Grand Island, NY, USA), 5% defined supplemental calf serum (HyClone, Life Technologies, Grand Island, NY, USA), 1% penicillin/streptomycin (Gibco, Life Technologies, Grand Island, NY, USA) and 1% L-glutamine (ThermoFisher Scientific, Cincinnati, NY, USA). Human fibroblasts grew in a medium containing DMEM (Gibco, Life Technologies, Grand Island, NY, USA), 10% FBS and 1% penicillin/streptomycin (all from Gibco, Life Technologies, Grand Island, NY, USA). Proteins were extracted from fibroblasts and myoblasts using radioimmunoprecipitation assay (RIPA) buffer resolved on the 4–20% polyacrylamide gel and analyzed by Western blot assay as described before [18]. RIPA buffer contains 25 mM tris HCl, pH 7.6; 150 mM sodium chloride, 1% Igepal CA-630 (NP-40), 1% sodium deoxycholate; 0.1% SDS.

### 4.6. Western Blot Analysis of Proteins

Prior to evaluating GSK3β levels in blood samples of patients with DM1, the sensitivity of the Western blot assay was tested using different amounts (from 0.5 to 10 ng) of purified recombinant GST-GSK3β. As shown in Appendix A—Figure A1A, this assay could detect 0.5 ng of the recombinant GSK3β (Abcam, Cambridge, UK) by Western blot analysis. Since the intensity of the GSK3β-containing band with 0.5 ng of protein is strong, it is expected that <0.5 ng of GSK3β could also be detected. Using these conditions of Western blot, the pre-experiment was performed to test if GSK3β is detectable in human PBMCs of DM1 patients. Different amounts of proteins (1, 10, 20 and 100 μg) from the PBMCs, isolated from whole blood samples of three patients with CDM1, were analyzed by Western blot analysis with antibodies to GSK3β. As shown in Appendix A—Figure A1B, the active GSK3β is detectable in 1–10 μg of total proteins from PBMCs of CDM1 patients. We used an enhanced Western blot protocol that involved pelleting PBMCs and incubating them with 2% sample buffer at 95 °C for 30 min. After heating, the samples were spun down at 10,000 rpm for 5 min, and supernatant was loaded onto gels. To examine GSK3β, THBS1, TGFβ and hnRNP-A3 in the human PBMCs; 10 μg of total proteins from PBMCs were separated by the 4–20% polyacrylamide gel electrophoresis; proteins were transferred onto a nitro cellulose membrane and probed with primary antibodies to phospho-GSK3β-Y216 (EpigenTek, New York, NY, USA), total GSK3β and β-actin (Santa Cruz Biotechnology Inc., Dallas, TX, USA), THBS1 (Proteintech Group, Inc., Rosemont, IL, USA), TGFβ (MilliporeSigma, Burlington, VT, USA) and hnRNP-A3 (ThermoFisher, Dallas, TX, USA) according to the manufactures’ suggestions overnight. TrueBlot (Rockland Immunochemicals, Inc., Pottstown, PA, USA) secondary antibodies were used for their high sensitivity for the Western blots. Protein signal quantification in Western blot analyses was performed by calculating the average band intensity adjusted to Coomassie-stained proteins based on two to three repeats contingent upon human material availability.

### 4.7. Statistical Analysis

In the muscle strength evaluation study, for each subject, the mean and variance of their right-hand grip strength was computed. A weighted linear regression model was fitted where the mean grip strength was modeled as a function of the CTG repeat length. The weight for each observation (subject mean) was the reciprocal of the subject’s variance. Similar analyses were conducted for left hand grip strength and right and left ankle dorsiflexion. An unweighted linear regression model was fitted for the 6MWT. Regression analyses were also applied to model GSK3β and THBS1 changes as a function of the CTG repeat length. These analyses were conducted using SAS ^®^ version 9.4 (SAS Institute Inc., Cary, NC, USA). No adjustment was made for multiple testing. To compare the GSK3β and THBS1 levels between the two patient groups, a two-tailed Student’s *t*-test was used. *p* values less than 0.05 were regarded as statistically significant.

## Figures and Tables

**Figure A1:**
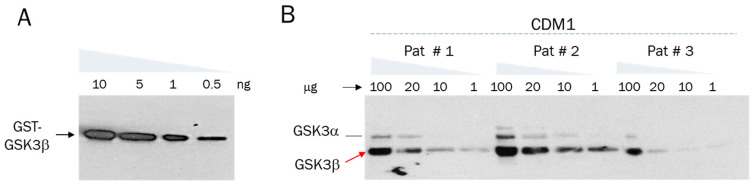
Sensitivity of GSK3β detection by Western blot assay. (**A**) Different amounts of the purified GST-GSK3β (0.5, 1, 5 and 10 ng) were probed with antibodies to GSK3β. (**B**) Different amounts of the PBMC proteins (1, 10, 20 and 100 μg) from three CDM1 patients were probed with antibodies to GSK3. Antibodies sc-7291 recognize both GSK3α and GSK3β.

## Data Availability

All primary data and protocols appear in the paper and Supplemental Materials. PBMC samples from some patients may not be available since small amounts of PBMCs were collected from 2 mL of whole blood. RNAseq data mentioned in the manuscript are not reported in this study and will be a part of a separate study. Any further reasonable data requests, which do not compromise the participants’ privacy, can be directed to the corresponding author.

## Supplementary Materials

The following supporting information can be downloaded at: https://www.mdpi.com/article/10.3390/ijms262110760/s1.

## Abbreviations

The following abbreviations are used in this manuscript:

6MWT	Six-minute walk test
CNS	Central Nervous System
CDM1	Congenital Myotonic Dystrophy 1
CUGBP1	CUG triplet repeat binding protein or CUGBP1 Elav-like family member 1), implicated in DM1
DM1	Myotonic Dystrophy type 1
DMEM	Dulbecco’s Modified Eagle Medium
DMPK	Myotonin Protein Kinase
FBS	Fetal Bovine Serum
GSK3β	Glycogen Synthase Kinase 3β
hnRNP-A3	Heterogeneous nuclear ribonucleoprotein A3
JDM1	Juvenile Myotonic Dystrophy 1
MBNL1	Muscleblind like splicing regulator 1, binding to CUG repeats, implicated in DM1
PBMCs	Peripheral blood mononuclear cells
TG	Tideglusib
THBS1	Thrombospondin 1
TGFβ	Transforming Growth Factor β
