# Supplementary material for: Elevated Levels of Active GSK3β in the Blood of Patients with Myotonic Dystrophy Type 1 Correlate with Muscle Weakness
Source: Int J Mol Sci. 2025 Nov 5;26(21):10760. doi: 10.3390/ijms262110760 (PMC12609377; doi:10.3390/ijms262110760)
Supplement: Supplementary file 1 [file ijms-26-10760-s001.zip › ijms-3936115-supplementary.pdf]

SUPPLEMENTAL MATERIALS

Supportive Data for Figure 1B, C, D and Figure 3. Immunoanalysis of active GSK3β in blood of patients with adult-onset DM1 vs controls (three repeats)

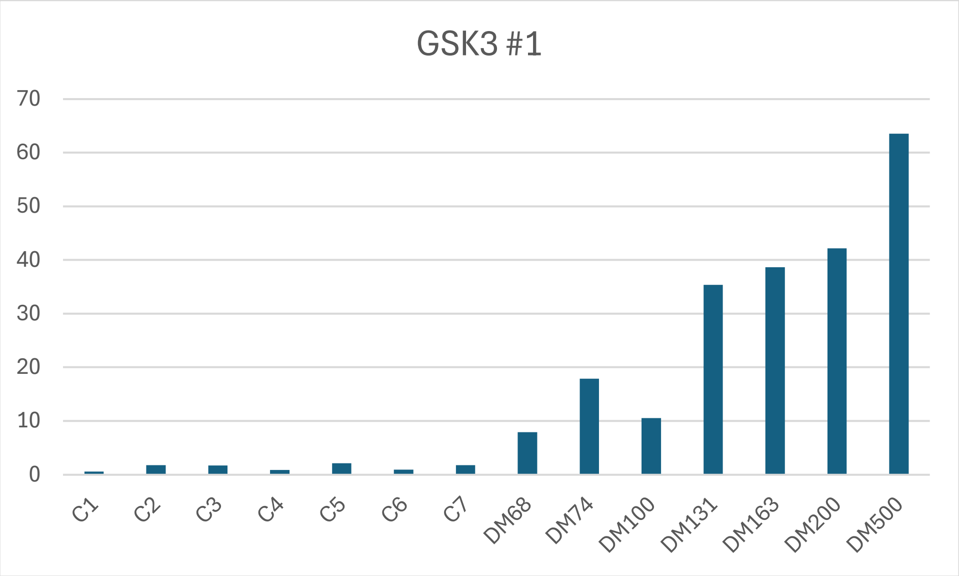

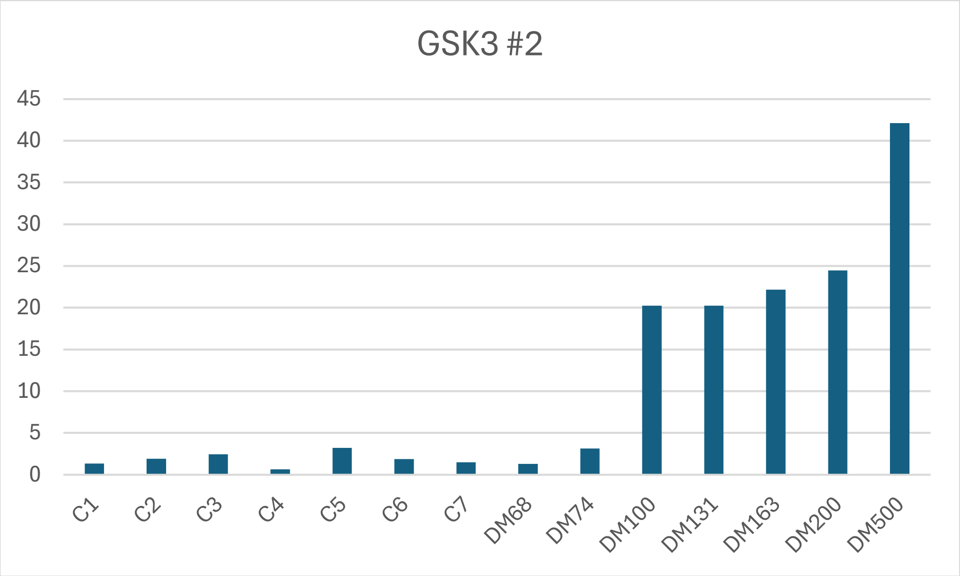

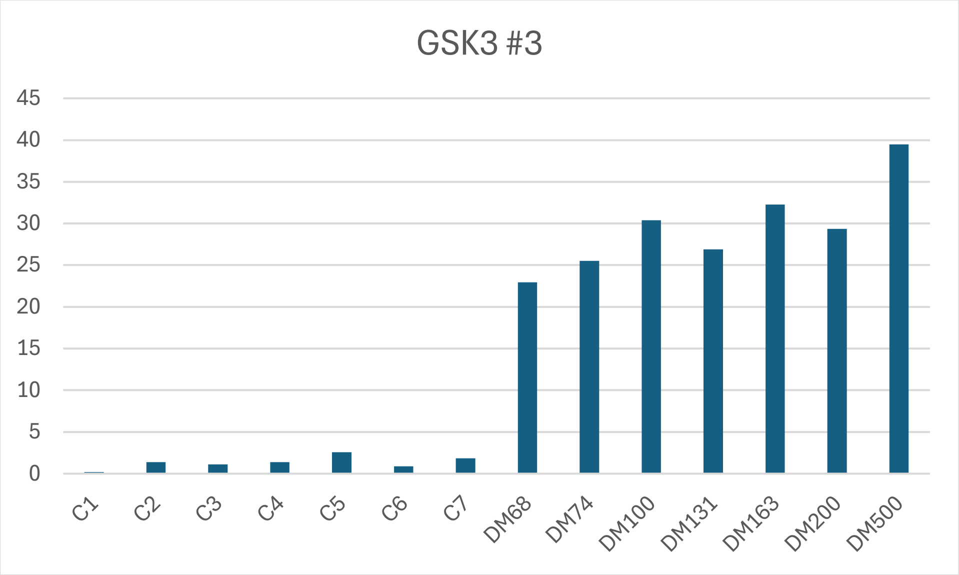

Figure S1

Supportive Data for Figure 1B, F and Figure 3. Immunoanalysis of THBS1 in blood of patients with adult-onset DM1 vs controls (three repeats)

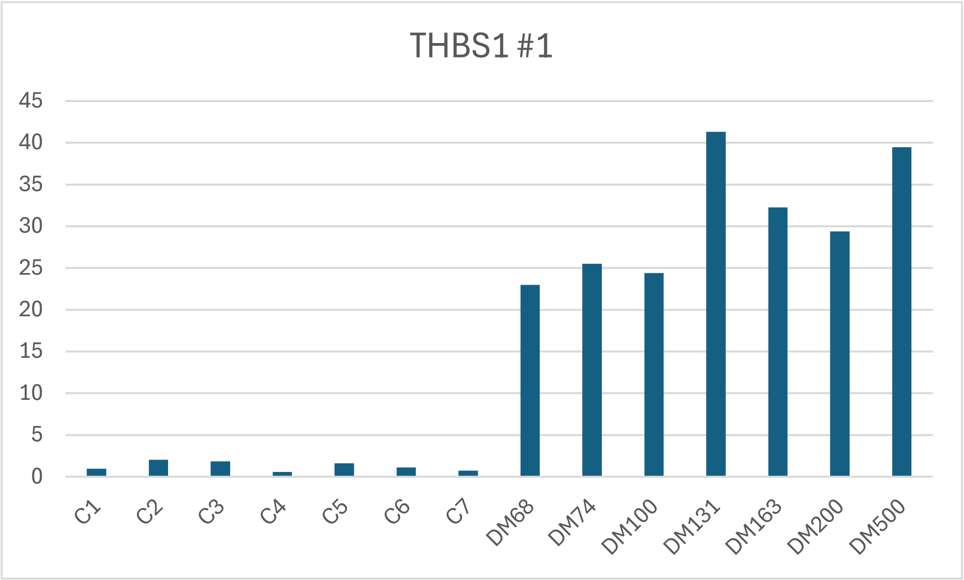

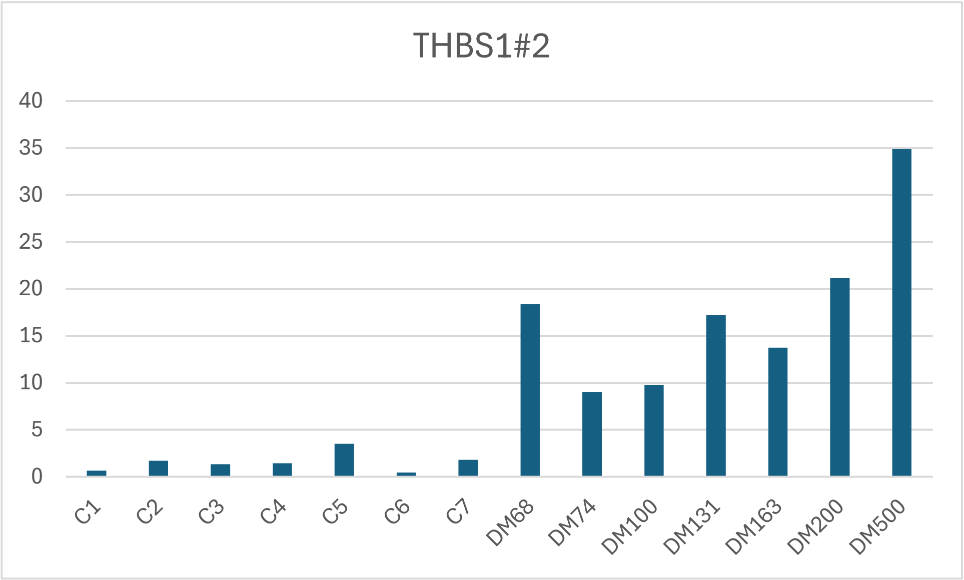

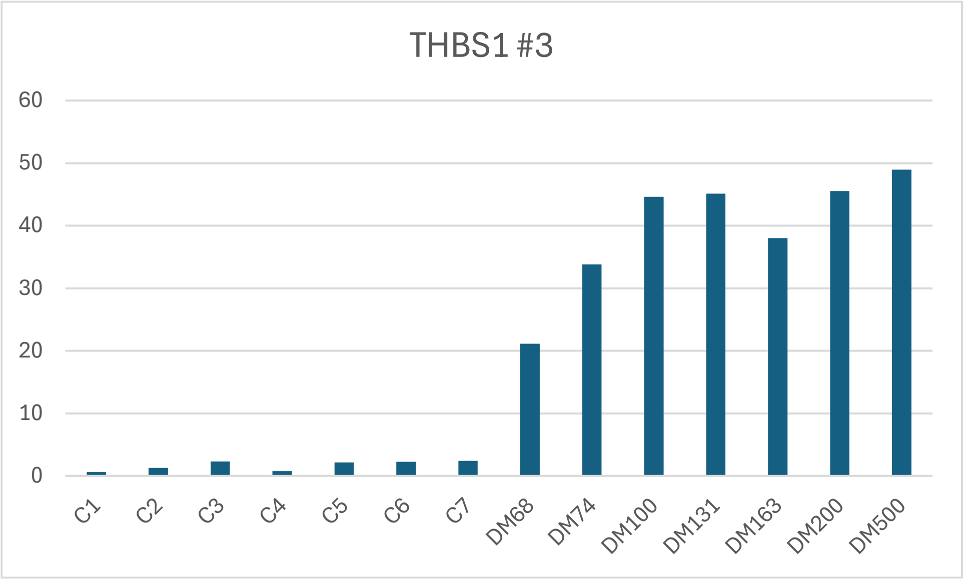

Figure S2

Supportive Data for Figure 4A, B. Immunoanalysis of active GSK3β in blood of patients with CDM1 and JDM1 vs controls (two repeats due to limited amounts of some blood samples from CDM1 patients)

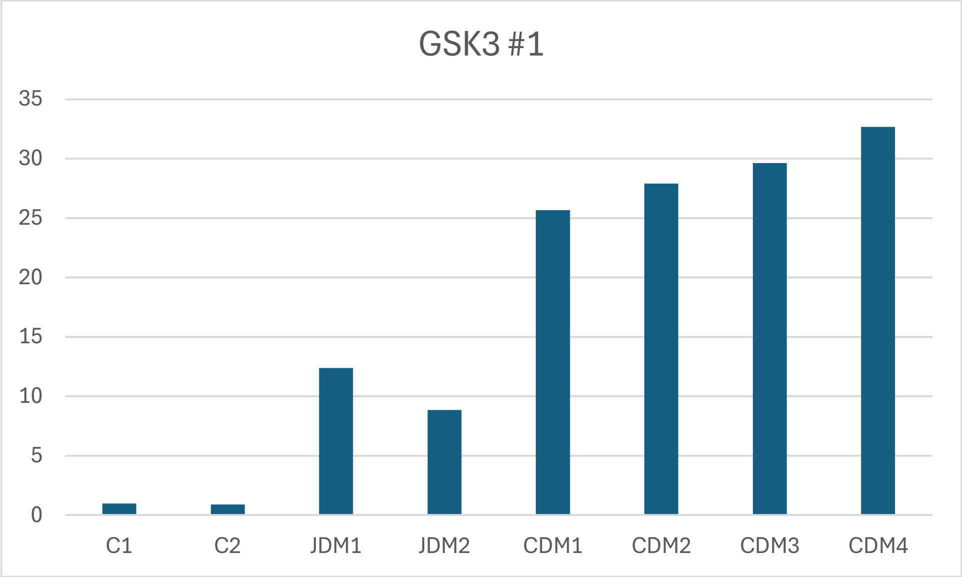

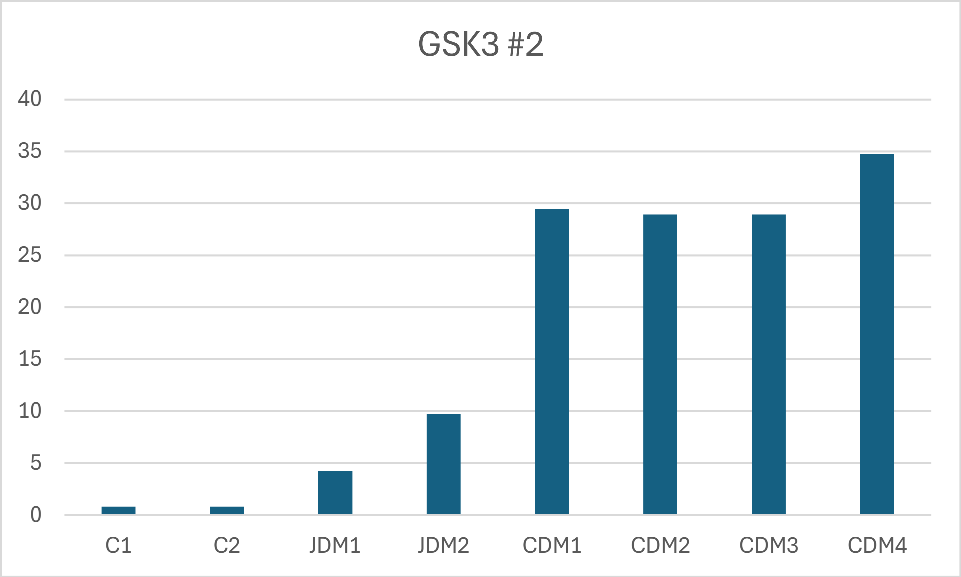

Figure S3
